# Optimization of Heart Block in the Left-Sided Whole Breast Radiation Treatments

**DOI:** 10.3389/fonc.2014.00342

**Published:** 2014-12-02

**Authors:** Ning J. Yue, Sharad Goyal, Joo Han Park, Sheri Jones, Xiaoting Xu, Atif Khan, Bruce G. Haffty, Ting Chen

**Affiliations:** ^1^Department of Radiation Oncology, Rutgers Cancer Institute of New Jersey, Rutgers-Robert Wood Johnson Medical School, Rutgers, The State University of New Jersey, New Brunswick, NJ, USA; ^2^Department of Radiation Oncology, The First Affiliated Hospital of Soochow University, Suzhou, China

**Keywords:** heart block optimization, left breast radiotherapy, breast cancer, intra-fractional motion, image processing

## Abstract

**Purpose**: Blocks have been used to protect heart from potential radiation damage in left-sided breast treatments. Since cardiac motion pattern may not be fully captured on conventional 3DCT or 4DCT simulation scans, this study was intended to investigate the optimization of the heart block design taking the cardiac motion into consideration.

**Materials and Methods**: Whole breast treatment plans using two opposed tangential fields were designed based on 4DCT simulation images for 10 left-sided breast cancer patients. Using an OBI system equipped to a Varian Linac, beam-eye viewed fluoroscopy images were acquired for each of the treatment beams after patient treatment setup, and the MLC heart blocks were overlaid onto the fluoroscopy images with an in-house software package. A non-rigid image registration and tracking algorithm was utilized to track the cardiac motion on the fluoroscopy images with minimal manual delineation for initialization, and the tracked cardiac motion information was used to optimize the heart block design to minimize the radiation damage to heart while avoiding the over-shielding that may lead to underdosing certain breast tissues.

**Results:** Twenty-three sets of fluoroscopy images were acquired on 23 different days of treatment for the 10 patients. As expected, heart moved under the influences of both respiratory and cardiac motion. It was observed that for 16 out of the 23 treatments, heart moved beyond the planed heart block into treatment fields and MLC had to be adjusted to fully block heart. The adjustment was made for all but one patient. The number of the adjusted MLC leaves ranged from 1 to 16 (mean = 10), and the MLC leaf position adjustment ranged from 2 to 10 mm (mean = 6 mm). The added heart block areas ranged from 3 to 1230 mm^2^ (mean = 331 mm^2^).

**Conclusion:** In left-sided whole breast radiation treatments, simulation CT (and 4DCT) based heart block design may not provide adequate heart protection for all the treatments. A fluoroscopy-based method has been developed to adaptively optimize the heart MLC block to achieve optimal heart protection.

## Introduction

Radiotherapy is an effective treatment modality for early-stage breast cancer (1–7). However, during the treatments, especially in left-sided patients, the heart inevitably receives a non-negligible amount of radiation doses. Taylor et al. estimated the cardiac doses of 358 patients received from breast cancer radiotherapy in Sweden during the period of time from the 1950s to the 1990s (8). They found that in this group of patients treated with relatively outdated technologies the mean heart dose varied from 0.1 to 23.6 Gy while the mean left anterior descending coronary artery dose varied from 0.1 to 46.3 Gy. They also reported that heart doses were significantly higher among the patients treated for left-sided breast cancer than for right-sided breast cancer (respectively, 5.1 and 1.8 Gy in the 1950s, 10.5 and 4.7 Gy in the 1970s, and 3.0 and 1.9 Gy in the 1990s). It is interesting and unsurprising to notice that the heart doses changed with the time period that was associated with technology advancement, indicating the important roles of radiotherapy technologies in the management and reduction of the heart doses. Many studies have shown that the heart dose received during the breast treatments can lead to long-term side effects and toxicities (9–15). Darby et al. conducted a population-based case–control study of major coronary events in 2168 women who underwent radiotherapy for breast cancer between 1958 and 2001 in Sweden and Denmark and concluded that exposure of the heart to ionizing radiation during breast cancer radiation treatment increases the subsequent rate of ischemic heart disease and the increase is proportional to the mean dose to the heart (9). A study by Nilsson et al. specifically investigated the distribution of coronary artery stenosis after radiation for breast cancer (11). They found that stenosis in mid and distal left anterior descending artery and distal diagonal increased in irradiated left-sided breast cancer and an association between irradiated high risk areas and stenosis in hot spots of radiation, indicating a direct link between radiation and location of coronary stenosis in breast cancer radiotherapy treatments. These more recent results expanded or confirmed the findings by other investigators (10, 12–15).

**Figure 1:**
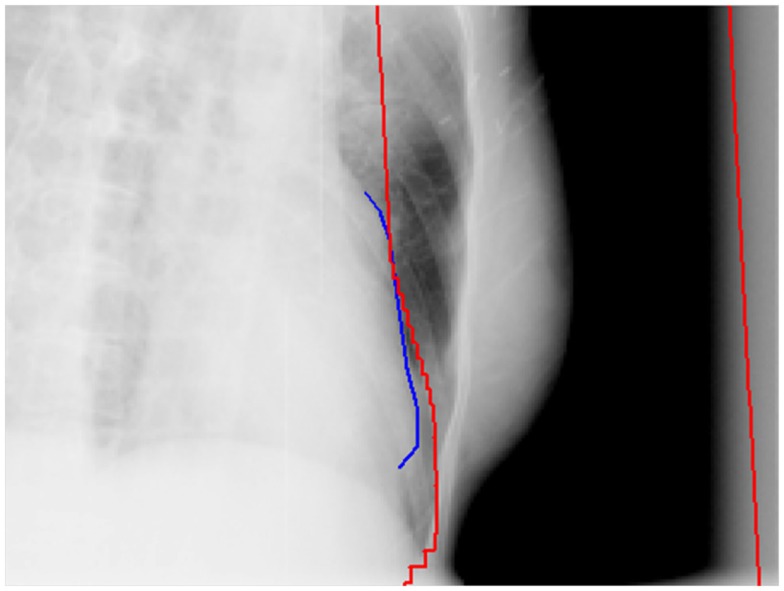
**An example of direct heart block in a tangential field of a whole breast radiation treatment**. The red lines are the field edges and the blue line is the heart outline on the side of the field in the beam’s eye view.

To minimize the potential cardiac toxicities from breast cancer radiation treatments, various breath control techniques were investigated for their control of the heart doses received in the breast radiation treatments (16–19). The breath-hold and breathing gating techniques have been demonstrated and clinically implemented to reduce the heart doses and irradiated cardiac volumes for left-sided breast cancer treatments. Another commonly adopted method is direct block of heart in the radiation treatment fields of breast (Figure 1). This method can also be used along with the breath control techniques for some patients if those techniques are deemed inadequately protecting heart from radiation.

Heart blocks are usually designed based on the heart shapes outlined on the simulation CT images or directly on the beam’s eye views of digitally reconstructed radiographs/regular radiographs of the radiation. However, since heart constantly moves, the heart outlines captured on the conventional CT images and radiographs may not reflect the full ranges of the motion. Although 4DCT may be used to analyze and incorporate the motion in the block design, it is often too slow to accurately estimate the heart motion. Furthermore, the heart block inevitably shields some breast tissues, which may require to be irradiated, from radiation, leading to potential target miss. The question remains on how to optimize a balance between minimal target miss and maximal heart block while incorporating heart beating motion. This study is to utilize an in-house developed fluoroscopy image rendering and registration algorithm to evaluate and optimize the heart block during left-sided whole breast irradiation treatments. The project was conducted under an IRB approved protocol.

## Materials and Methods

For left-sided breast cancer radiation treatments that require heart blocks, due to the nature of potential heart movement, the fluoroscopy imaging modality of the On-Board-Imaging (OBI) system equipped to linear accelerators can be used to dynamically check the appropriateness of the heart block designs at treatment. Ten patients were randomly selected from a pool of left-sided breast cancer patients who would receive the whole breast irradiation and required heart blocks. The whole breast treatments, using two opposed tangential fields, were planned based on 4DCT simulation images. In the plans, the heart blocks were manually designed and shaped with MLCs (Millennium 120 MLC, Varian Medical Systems, Palo Alto, CA, USA). The heart block designs took the considerations of heart locations on the 4DCT and clinical evaluation of the blocked breast tissues. For the 10 patients included in this study, the heart blocks were all such designed so that the block edges covered the heart borders reflected on the 4DCT average image sets.

Using an OBI system equipped to a Varian Linac, beam-eye viewed fluoroscopy images (for a duration of 10–15 s) were acquired for each of the treatment beams after patient treatment setup, and the MLC heart blocks were overlaid onto the fluoroscopy images with an in-house software package. A non-rigid image registration and tracking algorithm was utilized to track the cardiac motion on the fluoroscopy images with minimal delineation for initialization, and the tracked cardiac motion information was used to optimize the heart block design to minimize the radiation damage to heart while avoiding the over-shielding that may lead to underdosing certain breast tissues. To be consistent with the principle of heart block design in plans, the fluoroscopy-based heart block optimization was achieved when the MLC edges were adjusted to cover the heart borders imaged on the fluoroscopy.

A brief description of the non-rigid image registration and tracking algorithm as well as the MLC shape optimization principle is presented as follows.

### Motion modeling

Patient heart was initially delineated in fluoroscopy using the combination of CT to fluoroscopy image registration and was manually adjusted on the first frame of the fluoroscopy. The heart motion was then dynamically tracked in fluoroscopy using the registration propagation algorithm as briefly described below.

Given the initial heart delineation *C*, the registration between two fluoroscopy frames is based on an enhanced Demons algorithm, which uses the following equation of active force at point *i* ∈ *C*:

$$\begin{array}{llll} {\vec{f}}_{i}^{d} & =\left(m_{i}-s_{i}\right)\; & & \;\\ & \;\times \left(\frac{\vec{\nabla }s_{i}}{{∥\vec{\nabla }s_{i}∥}^{2}+a^{2}{\left(m_{i}-s_{i}\right)}^{2}}+\frac{\vec{\nabla }m_{i}}{{∥\vec{\nabla }m_{i}∥}^{2}+a^{2}{\left(m_{i}-s_{i}\right)}^{2}}\right)\; & & \; \end{array}$$

where *m* is the moving (target) frame, *s* is the static (source) frame, and *a* is a weighting parameter that controls the step size in the deformation. A constraint is defined as another force term $\overset{⇀}{f_{i}^{c}}$ to maintain the smoothness of the contour (heart surface).

$${\vec{f}}_{i}^{c}=\frac{1}{k}\sum_{j=1}^{k}f_{j}\exp\left(-\frac{{d^{2}}_{\mathrm{ij}}}{\sigma^{2}}\right)$$

where *f_j_* is the image force at a neighboring point *j* on the heart contour, and σ is the size of the neighborhood in which the smoothness factor will be effective.

The accumulative force driving the deformation can be expressed as:

$${\vec{f}}_{i}=\lambda_{c}{\vec{f}}_{i}^{c}+\lambda_{d}{\vec{f}}_{i}^{d},$$

where *λ_c_* and *λ_d_* are weights for the image force and the object constraint, respectively. We use empirical values that *λ_c_* = 0.25 and *λ_d_* = 0.75 for the calculation of the overall deformation force in the registration since these two values performed the best.

To improve the efficiency, the registration is conducted for cropped regions only. The size of the cropped region is automatically determined based on the delineation of the heart surface. Hierarchy strategy and frequency domain calculation are used to further speed up the registration process. After the registration, the motion vector (*d_x_*, *d_y_*, *d_z_*,) between corresponding pixels in different frames is calculated to generate a motion model so that the displacement at any point *i* in the heart contour on any two neighboring fluoroscopy frames can be expressed as:

$${\left(x,y,z\right)}_{j}^{i}={\left(d_{x}^{i},d_{y}^{i},d_{z}^{i}\right)}_{j,j+1}+{\left(x,y,z\right)}_{j+1}^{i},$$

where ${\left(x,y,z\right)}_{j+1}^{i}$ and ${\left(x,y,z\right)}_{j}^{i}$ are the positions of the same point on the heart contour in different fluoroscopy frame *j* + *1* and *j*, respectively. To retrieve the heart motion throughout the fluoroscopy, we propagate the registration to get the motion between any two arbitrary fluoroscopy frames *j* and *k* using:

$${\overset{⇀}{d}}_{\mathrm{jk}}=\left({\overset{⇀}{d}}_{j,j+1}+{\overset{⇀}{d}}_{j+1,k}+{\overset{⇀}{d}}_{j,k-1}+{\overset{⇀}{d}}_{k-1,k}\right)∕2.$$

The heart wall displacement with regard to the heart position in the first frame can be determined using the displacement map.

### MLC adjustment

The tracked cardiac motion was analyzed and taken into account for the adaptive optimization of the heart block design. The shape of the heart block was exported from the Eclipse (Varian) treatment planning system as the MLC file and loaded into our internally developed software. After the retrieval of heart motion, the maximal offset between the heart motion inside the treatment beam and the corresponding MLC leaf position is computed and used to adjust the MLC position in the original treatment plan to maintain full heart shielding.

In the heart block MLC position optimization process, if heart was adequately covered by the planned MLC positions, no MLC position adjustment was made; if it was found that heart was not adequately covered by the planned MLC, the MLC positions were adjusted so that the MLC edges covered the most infiltrating borders of heart into the corresponding field of radiation detected on the fluoroscopy images.

The optimized MLC heart blocks were checked for their appropriateness by an experienced radiation oncologist.

## Results

For the 10 patients, 23 sets of fluoroscopy images were acquired on 23 different days of treatment (Table 1). As expected, heart moved under the influences of both respiratory and cardiac motion. It was observed that for 16 out of the 23 treatments, heart moved beyond the planed heart block into treatment fields and MLC had to be adjusted to fully block heart. The adjustment was made for all but one patient, whose digital fluoroscopy was available for only one treatment. The number of the adjusted MLC leaves ranged from 1 to 16 (mean = 10), and the MLC leaf position adjustment ranged from 2 to 10 mm (mean = 6 mm). The added heart block areas ranged from 3 to 1230 mm^2^ (mean = 331 mm^2^). The results are summarized in Table 1.

**Table 1:** **Summary of heart block adjustments for the investigated left-sided breast cancer patient whole breast radiation treatments**.

Patient	No. of fluoro taken	Fluoro No.	Number of adjusted MLC leaves	Largest leaf adjustment (mm)	Total adjusted area (mm^2^)
A	2	1	0	0.0	0.0
		2	7	2.3	45.4
B	2	1	0	0.0	0.0
		2	1	3.7	3.1
C	2	1	0	0.0	0.0
		2	16	5.6	285.4
D	4	1	10	5.0	171.5
		2	7	3.0	61.4
		3	12	8.8	347.8
		4	12	8.5	369.2
E	4	1	6	2.0	35.1
		2	0	0.0	0.0
		3	0	0.0	0.0
		4	0	0.0	0.0
F	2	1	15	6.3	349.3
		2	9	4.8	157.4
G	1	1	0	0.0	0.0
H	2	1	6	3.2	61.8
		2	13	9.2	481.9
I	2	1	8	10.3	307.8
		2	9	7.0	227.7
J	2	1	16	9.3	1162.0
		2	16	9.9	1230.4

In the cases investigated in this study, the dose distributions were recalculated using the updated MLC positions. The dose coverage of the whole breast was compared to that of the original corresponding plan. No significant dose distribution difference was observed.

## Discussions and Conclusion

During whole breast radiation treatments, ideally the entire breast tissues should be irradiated and receive a therapeutic radiation dose. To minimize potential radiation damages to heart in the left-sided breast cancer treatments, heart blocks are added and their addition may compromise the irradiation of some breast tissues that fall under the blocks. This study only attempted to address the potential suboptimal heart protection, without trying to address the optimal balance between heart protection and breast tissue irradiation.

It is well known that patient organ motion (e.g., heart and respiration), in terms of motion frequency and magnitude, is very likely not exactly reproducible. The revised heart blocks, based on the fluoroscopy images acquired prior to the treatment, may not provide complete heart protection during the radiation beam-on time, if the organs do not exhibit the motion patterns as imaged on the fluoroscopy.

Dose distributions in the breast tissues, including both absolute and relative values, vary with beam field sizes. The changes of heart blocks at treatment will change the dose distributions and may introduce unexpected effects. As shown in Table 1, the observed mean area change was a little over 300 mm^2^. Given that typical tangential breast field size is over 20,000 mm^2^, it is reasonable to assume that for most cases, the heart block adjustment at treatment will have insignificant impact to the dose distributions with same machine outputs. However, in certain extreme cases, the block area change could be as large as over 1200 mm^2^ (Table 1), the impact on the dose distribution may not be trivial and may have to be taken into account if the heart block is to be changed.

The algorithm to track the motion and optimize the heart block is very fast and takes only a few seconds to complete the entire process. However, the acquisition of the fluoroscopic images, which includes rotating machine gantry and image acquisition itself, can take up to a few minutes. Therefore, the clinical implementation of the technique may add a few more minutes to the treatment time.

In conclusion, simulation CT (and 4DCT) based heart block design may not provide adequate heart protection for all the fractions in left-sided whole breast radiation treatments. A fluoroscopy-based method has been developed to adaptively optimize the heart MLC block to achieve optimal heart protection. On the other hand, additional study needs to be conducted to seek an optimal balance between protection of heart and assurance of entire breast tissue irradiation.

## Author Note

This project was presented as an oral presentation at the 55th annual AAPM meeting in August 2013, Indianapolis, IN, USA.

## Conflict of Interest Statement

The authors declare that the research was conducted in the absence of any commercial or financial relationships that could be construed as a potential conflict of interest.
